# Attitudes toward the SARS-CoV-2 Vaccine: Results from the Saudi Residents’ Intention to Get Vaccinated against COVID-19 (SRIGVAC) Study

**DOI:** 10.3390/vaccines9070798

**Published:** 2021-07-18

**Authors:** Sami H. Alzahrani, Mukhtiar Baig, Mohammed W. Alrabia, Mohammed R. Algethami, Meshari M. Alhamdan, Nabil A. Alhakamy, Hani Z. Asfour, Tauseef Ahmad

**Affiliations:** 1 Family Medicine Department, Faculty of Medicine, King Abdulaziz University, P.O. Box 80205, Jeddah 21589, Saudi Arabia; malhamdan1@kau.edu.sa; 2 Faculty of Medicine, King Abdulaziz University, Jeddah 21589, Saudi Arabia; mbbaig@kau.edu.sa; 3 Department of Medical Microbiology and Parasitology, Faculty of Medicine, King Abdulaziz University, Jeddah 21589, Saudi Arabia; mwalrabia@kau.edu.sa (M.W.A.); hasfour@kau.edu.sa (H.Z.A.); 4 Preventive Medicine and Public Health Resident, Ministry of Health, Jeddah 21577, Saudi Arabia; abo-reada-511@hotmail.com; 5 Department of Pharmaceutics, Faculty of Pharmacy, King Abdulaziz University, Jeddah 21589, Saudi Arabia; nalhakamy@kau.edu.sa; 6 Department of Epidemiology and Health Statistics, School of Public Health, Southeast University, Nanjing 210096, China; tahmad@seu.edu.cn

**Keywords:** vaccination, hesitancy, COVID-19, Saudi Arabia

## Abstract

Vaccine uptake could influence vaccination efforts to control the widespread COVID-19 pandemic; however, little is known about vaccine acceptance in Saudi Arabia. The present study aimed to assess the Saudi public’s intent to get vaccinated against COVID-19 and explore the associated demographic determinants of their intentions as well as the reasons for vaccine hesitancy. A cross-sectional, web-based survey was distributed to public individuals in Saudi Arabia between 25 December 2020 and 15 February 2021. Participants were asked if they were willing to get vaccinated, and the responses, along with demographic data were entered into a multinomial logistic regression model to assess the relative risk ratio (RRR) for responding “no” or “unsure” versus “yes”. Among 3048 participants (60.1% female, 89.5% Saudi), 52.9% intend to get vaccinated, 26.8% were unsure, and 20.3% refused vaccination. Vaccine hesitancy was significantly higher among females (RRR = 2.70, *p* < 0.0001) and those who had not been recently vaccinated for influenza (RRR = 2.63, *p* < 0.0001). The likelihood was lower among Saudis (RRR = 0.49, *p* < 0.0001), those with less than a secondary education (RRR = 0.16, *p* < 0.0001), perceived risks of COVID-19, and residents of the southern region (RRR = 0.46, *p* < 0.0001). The most often cited reasons for hesitancy were short clinical testing periods and concerns about adverse events or effectiveness. Vaccine hesitancy is mediated by many demographic factors and personal beliefs. To address vaccine-related concerns and amend deeply rooted health beliefs, communication should provide transparent information.

## 1. Introduction

The widespread new strains of the coronavirus (SARS-CoV-2) have represented a major health burden globally. As of 28 February 2021, the novel coronavirus disease (COVID-19) has affected more than 113 million individuals and caused more than 2.5 million deaths worldwide [1]. In Saudi Arabia, more than 377,000 confirmed cases and more than 6400 COVID-19-related deaths have been reported across the Kingdom [2]. Globally, COVID-19 has caused widespread havoc, and the consequences have impacted people on financial, social, mental, and personal levels [3,4].

Indeed, Saudi Arabia is an important destination for millions of Muslims for Umrah and Hajj; therefore, it was among the first countries to implement strict precautionary measures to control the spread of the epidemic [5]. The success of public health efforts in different regions will largely depend on providing acquired immunity to a considerable proportion of the population, which has been ideally estimated at 67% [6]. Although the duration and degree of protection from vaccines remains enigmatic, widespread vaccination seems to be the most effective method of controlling the pandemic. The development of a COVID-19 vaccine has brought a glimmer of hope to the world. Vaccination not only decreases the incidence of disease but also protects non-immunized people in the general population [7].

However, vaccine acceptance and trust in the effectiveness and safety of immunization will play a central role in whether we attain the ideal coverage required to reach so-called herd immunity. Early data from European countries (April 2020) have indicated that the hesitancy regarding a prospective vaccine was prevalent among 26% of adults, and approximately the same proportion of hesitant individuals has also been reported in the United States [8]. Furthermore, a subsequent cross-sectional investigation carried out in July 2020 showed that vaccine rejection was reported among about one-third of adult populations [9]. Notably, there were specific demographic variations in the patterns of vaccine acceptance, with a greater likelihood of vaccine refusal among females and those with low incomes and low levels of education, as well as those who had not been vaccinated against influenza in the preceding year [9,10,11].

Based on the aforementioned observations, it is necessary to understand individuals’ attitudes about vaccination and identify the barriers that may affect their decisions in the context of the COVID-19 vaccine. As of 3 March 2021, more than 295,000 individuals have received the COVID-19 vaccination in Saudi Arabia [2]. To the best of our knowledge, only one national study has been conducted so far to assess participants’ willingness to get the vaccine [12]; the study involved four major cities and was carried out before the initiation of the vaccination program. With the increased body of data regarding the new vaccine in the media and from other information sources, perceptions and attitudes of the public might have changed [13]. Thus, the intention to receive the vaccine should be a matter of ongoing research. In the present study, we aimed to explore participants’ willingness to get vaccinated against COVID-19. Additionally, we assessed the demographic determinants of their intentions and the reasons for their vaccine hesitancy.

## 2. Materials and Methods

### 2.1. Participants and Survey Administration

The Saudi Residents’ Intention to Get Vaccinated Against COVID-19 (SRIGVAC) study was a cross-sectional survey of adults residing in Saudi Arabia. Participants were asked to fill out a structured questionnaire on a specifically designated platform (Google Forms) between 25 December 2020 and 15 February 2021. The questionnaire was adapted from previously published studies [9,14,15] and then circulated electronically using various social media platforms. The first screen informed potential participants about the aims of the survey and presented an informed consent notice. They were then advised that by clicking on the accept button, they were agreeing to the informed consent and could continue on to complete the survey.

The questionnaire was in English, but our participants were mostly Arabic speaking, so two bilingual translators handled the bidirectional translation. The questionnaire was then modified accordingly to improve respondents’ comprehension while maintaining the content and meaning. A pilot test was conducted with 50 people from the general population to check the questionnaire’s understandability, and it was then further modified accordingly. Questionnaire reliability was 82% according to Chronbach’s alpha. Two senior faculty members and a medical educationist reviewed the questionnaire’s construct and content validity, and it was further changed as suggested. We employed the snowball technique for data collection because of the COVID-19 restrictions. The calculated sample size was 770, and we further inflated that number to get valid and generalizable results.

### 2.2. Measures

Participants’ intentions regarding vaccination were measured using the item “If the COVID-19 vaccine became available in your country and was provided to you by the government free of charge, would you get vaccinated?” The responses were collected on a five-point Likert scale of strongly disagree, disagree, unsure, agree, and strongly agree. Positive responses (agree and strongly agree) and negative responses (disagree and strongly disagree) were then combined into yes and no categories for the subsequent analysis. Reasons for vaccination hesitancy were assessed using 11 items related to the vaccine itself (adverse events, lack of efficacy, etc.), lack of trust in pharmaceutical companies (in manufacturing, vaccine development, and clinical testing), or misperceptions about COVID-19 infection (belief that it is a harmless disease or having a preference for natural immunity). Data related to participant characteristics were also collected, including age, gender, nationality, household size, geographic location, education level, monthly income, and employment status. Additionally, the medical histories of chronic illness or COVID-19 infection were collected from respondents, and any history of COVID-19 infections among friends and family were also collected. For the geographic location variable, participants had to initially select a response from 13 available provinces. However, the responses were categorized into the five main regions of the central region (Riyadh, Ha’il, and Qassim Provinces), northern region (Tabuk, Al-Jouf, and the Northern Borders Provinces), southern region (Najran, Asir, and Jazan Provinces), eastern region (the Eastern Province), and western region (Mecca, Medina, and Al-Bahah Provinces). The participants were also asked whether they had received an influenza vaccine within the past year. Finally, respondents were also asked about how they perceive the risk of COVID-19 on both personal and community levels.

### 2.3. Statistical Analysis

Statistical Package for Social Sciences software version 26.0 (IBM, Inc., Armonk, New York, NY, USA) was used to perform the statistical analysis. Demographic and clinical characteristics of the participants and their perceived risks of COVID-19 were summarized as frequencies and percentages. Univariate unadjusted associations between such characteristics and the intention to get vaccinated were assessed using Pearson’s chi-square test. The adjusted associations were explored by performing multinominal logistic regression, which is used to assess the predictors of an outcome variable of two or more categories to a reference category. The dependent variable was participants’ intentions to receive the vaccine, and the reference category was the “yes” response. Participants’ characteristics that were found to be statistically significant in the univariate analysis were only included in the final regression model as potential predictors. The obtained exponentiated coefficients were interpreted as relative risk ratios (RRRs) and their respective 95% confidence intervals (95%CIs). Statistical significance was considered at *p* < 0.05.

## 3. Results

### 3.1. Demographic Characteristics

The responses of 3.091 participants were initially recorded. However, some records were excluded due to either a lack of primary outcomes (19 records) or reporting invalid age values (<18 years, 24 records). Therefore, the responses of 3048 participants were ultimately included in the subsequent analysis. The majority of respondents were aged 18–44 years (85.1%) and were Saudis (89.5%). More than half of the population under study were females (60.1%) residing in the western region (57.0%) and were currently employed (52.9%, Table 1). Of the latter group, 305 and 491 respondents declared that they were working completely or partially remotely, representing 18.9% and 30.5% of the working population, respectively.

### 3.2. Clinical Characteristics and the Perceived Risk of COVID-19

In general, less than half of the respondents (41.1%) had received a flu shot or flu spray within the last year. Of note, 580 respondents (19.0%) declared a history of chronic illness. Additionally, 349 respondents (11.5%) had experienced COVID-19, while the disease had affected family members and friends or coworkers of 32.3% and 66.0% of the participants, respectively. Regarding the perceived risk of COVID-19, less than two-thirds of the participants perceived the disease as representing a low to moderate risk to the individual (63.4%) and others in the community (62.3%, Table 2).

### 3.3. Vaccination Intention and Associated Factors

Overall, 1612 participants (52.9%) indicated that they would receive the SARS-CoV-2 vaccine as soon as it becomes available, 817 (26.8%) were unsure about getting vaccinated, and 619 (20.3%) revealed that they would not receive the vaccine. Unadjusted univariate association analysis showed that the intent to get vaccinated differed significantly by gender (*p* < 0.0001), nationality (*p* < 0.0001), educational attainment (*p* = 0.010), and region (*p* < 0.0001, Table 1). Furthermore, compared to their counterparts, vaccination intention was higher among participants who had received an influenza shot as well as those with relatively higher perceived risks for COVID-19 on the personal and community levels (Table 2).

The results of the adjusted multivariate regression analysis are demonstrated in Table 3. Females had less than threefold higher relative likelihood of not intending to receive the vaccine compared to males (RRR = 2.70, 95%CI, 2.18 to 3.36, *p* < 0.0001), and the relative likelihood remained significant for “unsure” responses (RRR = 1.69, 95%CI, 1.41 to 2.02, *p* < 0.0001). In addition, participants who had not received an influenza vaccine had a nearly threefold higher relative likelihood of providing “no” responses (RRR = 2.63, 95%CI, 2.12 to 3.25, *p* < 0.0001) and a nearly twofold higher relative likelihood of providing “unsure” responses (RRR = 1.88, 95%CI, 1.57 to 2.24, *p* < 0.0001).

Conversely, the relative likelihood of vaccine refusal was 84% lower among individuals with less than secondary education compared to individuals with post-graduate degrees (RRR = 0.16, 95%CI, 0.05 to 0.57, *p* = 0.005). Saudis had a 51% lower relative likelihood of not receiving the vaccine (RRR = 0.49, 95%CI, 0.36 to 0.66, *p* < 0.0001), and people residing in the southern region were less likely to be hesitant (RRR = 0.46, 95%CI, 0.30 to 0.69, *p* < 0.0001) or uncertain about vaccination (RRR = 0.61, 95%CI, 0.43 to 0.87, *p* = 0.006) than those residing in the central region. Finally, people with low to high perceived risk of COVID-19 had 37–55% lower relative likelihood of responding “no” regarding their vaccination intent (Table 3).

### 3.4. Reasons for No or Not Sure Responses Regarding Participant’s Intention to Get Vaccinated

**Figure 1:**
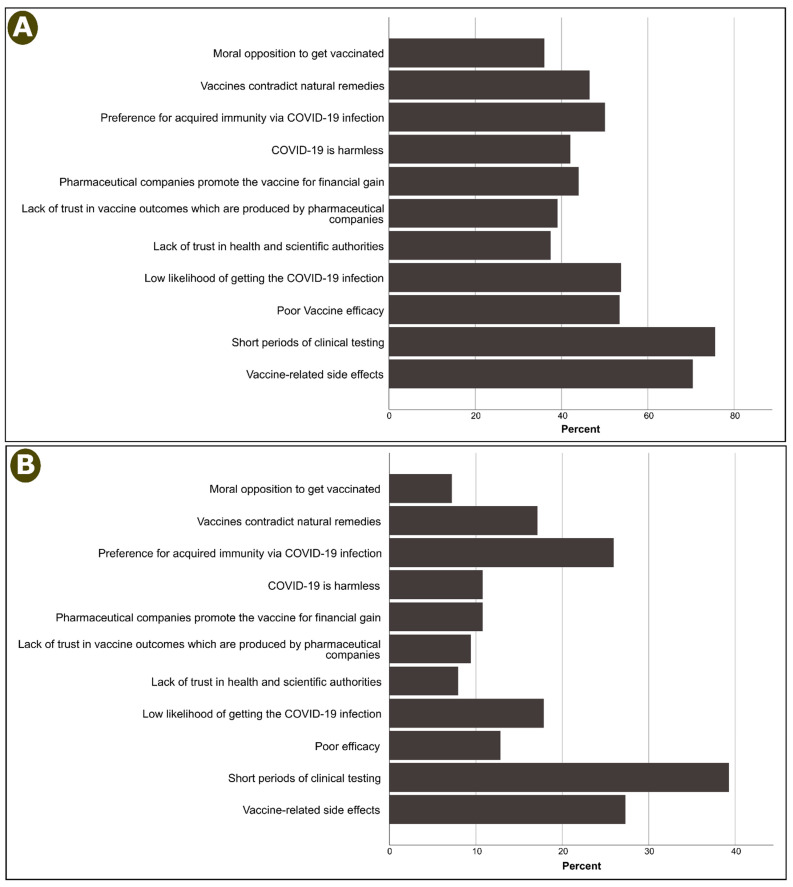
Reasons for hesitancy among participants who answered (**A**) no or (**B**) unsure regarding their intention to get vaccinated.

The reasons given by participants who replied no or unsure with regard to getting vaccinated are provided in Figure 1, and more detailed descriptive statistics are available in the Supplementary Materials (Tables S1 and S2). Short clinical testing was the most frequently given reason for rejecting vaccination, as indicated by 75.6% of those who provided no responses. This was followed by concerns about vaccine-related adverse events (70.4%), and then a self-perception as having a poor chance of contracting the disease and a perceived lack of vaccine efficacy (53.5%, Figure 1A).

Among the unsure participants, the most common reasons given for refusing vaccination were short clinical testing period (39.3%), vaccine side effects (27.3%), and preference for acquired immunity via contracting the COVID-19 infection (25.9%, Figure 1B).

## 4. Discussion

Vaccination is the mainstay public health approach for preventing the spread of infectious diseases; however, vaccines’ effectiveness relies on their use. Such an observation can be confirmed by the resurgence of measles and pertussis outbreaks due to widespread anti-vaccine attitudes in recent decades [16,17]. Therefore, vaccination efforts can be undermined by negative attitudes that mediate vaccine refusal, which would threaten the ultimate goal of herd immunity [18]. The present study showed that 47.1% of adults in Saudi Arabia were unsure or unwilling to get the COVID-19 vaccination, whereas only 52.9% of individuals intended to get vaccinated. Females and individuals who had not received a flu vaccine within the past year were more likely to refuse vaccination. Conversely, Saudis, participants with a perception of low to high risk of COVID-19, and those who had been vaccinated against influenza were more likely to intend to vaccinate.

The results of the present analysis revealed interesting findings. Vaccine uptake is generally lower in Saudi Arabia than what is reported in other countries. For instance, recent cross-sectional investigations indicated that the proportion of the populations that intended to vaccinate was 91.3% in China [19], 79.0% in the United Kingdom [20], 76.5% in Australia [21], and 53.6–62.2% in the United States [22,23]. Similarly, recent systematic reviews and meta-analyses have shown that acceptance rates were highest in China and Southeast Asia and were lowest in distinct Arab countries (Kuwait and Jordan) and some European countries, such as Italy and Russia [24,25]. In Saudi Arabia, another Arabic-speaking country, Al-Mohaithef and Padhi [12] conducted a web-based survey among 992 adults residing in four major cities, and the authors found that 62.2% of participants would be extremely or somewhat likely to get vaccinated. The lower rate of vaccine acceptance in our study indicates a declining trend in participants’ intentions.

Similarly, Daly and Robinson [23] also found a significant decline in vaccine acceptance, from 71% to 53.6% during the period between April and October 2020 in the United States. Interestingly, in a large web-based study of public individuals, Loomba et al. [26] showed that 54.1% and 42.5% of participants from the United States and the United Kingdom, respectively, would “definitely” get the vaccination, but those rates have dropped by 6.2% and 6.4%, respectively, after exposure to online misinformation about the vaccine. This indicates that the significant temporal decline in vaccination uptake might be explained by exposure to misleading information, conspiracy theories, and other false reports being spread online via social media platforms. Online anti-vaccine movements have already been significantly influencing public individuals’ perceptions and attitudes toward vaccinations of all types in the United States [27]. Nevertheless, little is known about such effects in the Saudi community. Therefore, the determinants of the impact of misleading information on individuals’ perceptions should be investigated in future studies in order to reduce vaccine refusal rates.

To some extent, the general public’s concerns regarding vaccine safety were significant, as shown in our study. Similarly, the top reasons for vaccination refusal in the earlier study in Saudi Arabia [12] included vaccine-related adverse events, concerns about allergic responses, worries about vaccine effectiveness, and preferences for acquired immunity through infection. As could be expected, the vaccines have been developed at unprecedented speeds, with a quick start and multiple development steps being carried out simultaneously before verifying successful outcomes in other steps [28]. This pandemic paradigm of vaccine development has raised concerns regarding the effectiveness and safety of vaccines in the context of short clinical testing times and the lack of reliable long-term outcomes that ensure substantial vaccine acceptance. Hence, short clinical testing and vaccine-related safety concerns were the major drivers of vaccine hesitancy in our analysis.

Of note, the lack of clinical testing among distinct populations might also mediate vaccine refusal. For example, hesitancy may be apparent due to the lack of evidence-based data regarding the benefits and risks of COVID-19 vaccine among patients with benign or malignant hematological conditions who would potentially exhibit an altered immune response to SARS-CoV-2 vaccines [29]. Similarly, the immune response in patients on hemodialysis is usually characterized by a reduced function of the innate and adaptive immunity, which may lead to hyporesponsiveness to vaccines [30]. Additionally, vaccine responsiveness is unknown among children and adolescents, particularly those with chronic conditions, such as obesity, disability, and chronic diseases [31]. These challenges and uncertainties underline the importance of raising the public’s awareness regarding the vaccine until reliable evidence has been established.

Additionally, vaccine refusal was predicted by distinct demographic groups. Females were more likely to oppose vaccination, which is in agreement with other studies [32,33,34]. Callaghan et al. [33] showed that females refused the vaccine due to concerns about vaccine safety and effectiveness. In contrast, hesitant males were more likely to express financial concerns (the cost of the prospective vaccine). People in the southern region were more likely to accept vaccination than those living in central region provinces. The central region includes Saudi Arabia’s capital Riyadh, which, like most capital cities, is highly developed. The literacy rate is very high in Riyadh, and thus our results indicate that educated people have more questions about the vaccine’s efficacy because of easy access to the media. Even after the emergence of different strains of the coronavirus, educated people have continued to harbor more doubts. It could be one of the plausible reasons for less acceptance of vaccination in this region. Intriguingly, getting an influenza vaccination was a significant predictor of getting vaccinated against COVID-19, which is consistent with the fact that past behavioral intentions can act as major drivers of future behavior across several health areas [35,36,37]. Similarly, the perceived risk of COVID-19 has played an important role in participants’ intentions, and this is in accord with previous studies demonstrating that the emotional side of risk perception had guided decisions to be vaccinated against influenza [38,39].

Based on these findings, public health experts and decision-makers need to adopt robust strategic plans to encourage vaccine uptake. It is also necessary to ensure that accurate information is communicated to the public through authentic sources who provide such information transparently and correctly. For instance, to address the existing concerns about vaccination, health care providers and the Ministry of Health could convey CDC recommendations using targeted conversations to address public vaccination concerns. In this way, inaccurate health information on social media platforms could be corrected, and data about vaccine effectiveness and safety would be publicly available to individuals. This would eventually promote confidence in the information and be supportive of individuals’ decisions regarding vaccination.

The present study employed the largest sample of the Saudi public so far. This might address the rising demand for relevant studies to assess public attitudes in the Middle East [25]. Additionally, we have provided insight into respondents’ intentions following the official approval of a number of vaccines, which might fill the gap of knowledge regarding the real community response to vaccines already in place and the temporal changes in individuals’ attitudes. However, the inherent limitations of a cross-sectional survey remain problematic, where the reporting bias might have affected outcomes. Additionally, the web-based method might result in different patterns of responses than would be received in a face-to-face design. Moreover, the representation from all five regions of the Kingdom was not equal.

## 5. Conclusions

In conclusion, about half of Saudis are unwilling or undecided about getting the COVID-19 vaccine, representing a significant public health threat and impediment to the goal of attaining herd immunity. Specific demographic groups were more likely to intend to vaccinate, such as males, Saudis, individuals with less than a secondary education, residence in the southern region, and individuals with perceived risks of COVID-19. Furthermore, participants who had received the influenza vaccine within the past year were more likely to accept the COVID-19 vaccine. Understanding why individuals may express hesitancy about vaccination is a key factor in designing targeted programs by stakeholders and decision-makers in the national health care system. More specifically, scientific data should be transparently and correctly conveyed to vaccine-hesitant populations to counter the misleading information that might be provided via inauthentic platforms.

## Figures and Tables

**Table 1:** Demographic characteristics of the participants and their distribution according to intention to be vaccinated (*n* = 3048).

Parameter	Category	N (%)	Intention to Be Vaccinated, *n* (%)			*p*
			Yes (*n* = 1612)	Unsure (*n* = 817)	No (*n* = 619)	
Age	18–29 y	1331 (43.7)	713 (53.6)	361 (27.1)	257 (19.3)	0.079
	30–44 y	1261 (41.4)	638 (50.6)	348 (27.6)	275 (21.8)	
	45–59 y	409 (13.4)	228 (55.7)	99 (24.2)	82 (20)	
	≥60 y	47 (1.5)	33 (70.2)	9 (19.1)	5 (10.6)	
Gender	Male	1217 (39.9)	779 (64)	284 (23.3)	154 (12.7)	<0.0001
	Female	1831 (60.1)	833 (45.5)	533 (29.1)	465 (25.4)	
Education level	<Secondary education	42 (1.4)	34 (81)	5 (11.9)	3 (7.1)	0.010
	Secondary	302 (9.9)	149 (49.3)	80 (26.5)	73 (24.2)	
	University	2104 (69)	1118 (53.1)	567 (26.9)	419 (19.9)	
	Post-graduate	600 (19.7)	311 (51.8)	165 (27.5)	124 (20.7)	
Nationality	Saudi	2729 (89.5)	1468 (53.8)	738 (27)	523 (19.2)	<0.0001
	Non-Saudi	319 (10.5)	144 (45.1)	79 (24.8)	96 (30.1)	
Current employment status	Employed—Government	1157 (38)	622 (53.8)	311 (26.9)	224 (19.4)	0.320
	Employed—Private/self-employed	530 (17.4)	285 (53.8)	127 (24.0)	118 (22.3)	
	Student	581 (19.1)	314 (54)	149 (25.6)	118 (20.3)	
	Not working	780 (25.6)	391 (50.1)	230 (29.5)	159 (20.4)	
Monthly income (SAR)	<3000	1220 (40)	641 (52.5)	332 (27.2)	247 (20.2)	0.067
	3000–10,000	851 (27.9)	426 (50.1)	228 (26.8)	197 (23.1)	
	>10,000–25,000	856 (28.1)	470 (54.9)	227 (26.5)	159 (18.6)	
	>25,000	121 (4)	75 (62)	30 (24.8)	16 (13.2)	
Household size	1–3	495 (16.2)	260 (52.5)	122 (24.6)	113 (22.8)	0.243
	4–6	1402 (46)	728 (51.9)	392 (28)	282 (20.1)	
	7–9	818 (26.8)	430 (52.6)	227 (27.8)	161 (19.7)	
	≥10	333 (10.9)	194 (58.3)	76 (22.8)	63 (18.9)	
Geographic region of residency	Western	1738 (57)	916 (52.7)	482 (27.7)	340 (19.6)	<0.0001
	Eastern	294 (9.6)	154 (52.4)	76 (25.9)	64 (21.8)	
	Northern	193 (6.3)	93 (48.2)	54 (28)	46 (23.8)	
	Southern	329 (10.8)	217 (66)	71 (21.6)	41 (12.5)	
	Central	494 (16.2)	232 (47)	134 (27.1)	128 (25.9)	

SAR = Saudi Riyal.

**Table 2:** Medical histories and experiences with COVID-19 among participants and their distribution by intention to be vaccinated (*n* = 3048).

Parameter	Category	N (%)	Intention to Be Vaccinated, *n* (%)			*p*
			Yes (*n* = 1612)	Not Sure (*n* = 817)	No (*n* = 619)	
History of a chronic disease	No	2468 (81)	1303 (52.8)	673 (27.3)	492 (19.9)	0.367
	Yes	580 (19)	309 (53.3)	144 (24.8)	127 (21.9)	
History of COVID-19	No	2699 (88.5)	1430 (53)	736 (27.3)	533 (19.7)	0.061
	Yes	349 (11.5)	182 (52.1)	81 (23.2)	86 (24.6)	
History of COVID-19 among family members	No	2064 (67.7)	1092 (52.9)	561 (27.2)	411 (19.9)	0.66
	Yes	984 (32.3)	520 (52.8)	256 (26)	208 (21.1)	
History of COVID-19 among friends	No	1035 (34)	543 (52.5)	271 (26.2)	221 (21.4)	0.571
	Yes	2013 (66)	1069 (53.1)	546 (27.1)	398 (19.8)	
Recently received an influenza vaccine	No	1794 (58.9)	798 (44.5)	535 (29.8)	461 (25.7)	<0.0001
	Yes	1254 (41.1)	814 (64.9)	282 (22.5)	158 (12.6)	
Perception of risk of COVID-19 to the individual	No	376 (12.3)	37 (38.9)	9 (9.5)	49 (51.6)	<0.0001
	Low to Moderate	1931 (63.4)	967 (50.9)	551 (29)	381 (20.1)	
	High to very high	741 (24.3)	608 (57.7)	257 (24.4)	189 (17.9)	
Perception of risk of COVID-19 to others	No	95 (3.1)	164 (43.6)	75 (19.9)	137 (36.4)	<0.0001
	Low to Moderate	1899 (62.3)	1034 (53.5)	556 (28.8)	341 (17.7)	
	High to very high	1054 (34.6)	414 (55.9)	186 (25.1)	141 (19)	

**Table 3:** Predictors of providing “no” and “unsure” responses regarding participants’ intent to get vaccinated.

Parameter	Category	Intent to Be Vaccinated			
		No vs. Yes		Not Sure vs. Yes	
		RRR (95% CI)	*p*	RRR (95% CI)	*p*
Gender	Female	2.70 (2.18–3.36)	<0.0001	1.69 (1.41–2.02)	<0.0001
	Male	Ref		Ref	
Nationality	Saudi	0.49 (0.36–0.66)	<0.0001	0.85 (0.63–1.15)	0.304
	Non-Saudi	Ref		Ref	
Educational level	<Secondary education	0.16 (0.05–0.57)	0.005	0.26 (0.1–0.68)	0.006
	Secondary	0.92 (0.63–1.34)	0.658	0.92 (0.66–1.29)	0.632
	University	0.84 (0.65–1.09)	0.183	0.88 (0.7–1.1)	0.256
	Post-graduate	Ref		Ref	
Geographic location	Western	0.78 (0.6–1.01)	0.061	0.93 (0.73–1.19)	0.576
	Eastern	0.83 (0.57–1.23)	0.356	0.87 (0.61–1.24)	0.445
	Northern	0.99 (0.64–1.53)	0.966	1.00 (0.66–1.49)	0.982
	Southern	0.46 (0.30–0.69)	<0.0001	0.61 (0.43–0.87)	0.006
	Central	Ref		Ref	
Received the influenza vaccine recently	No	2.63 (2.12–3.25)	<0.0001	1.88 (1.57–2.24)	<0.0001
	Yes	Ref		Ref	
Personal risk of COVID-19	High to very high	0.59 (0.35–0.99)	0.046	1.05 (0.79–3.09)	0.060
	Low to Moderate	0.45 (0.26–0.79)	0.006	1.68 (0.76–3.7)	0.200
	No	Ref		Ref	
Risk of COVID-19 to others	High to very high	0.52 (0.38–0.7)	<0.0001	1.16 (0.85–1.58)	0.356
	Low to Moderate	0.63 (0.43–0.91)	0.014	1.16 (0.8–1.67)	0.441
	No	Ref		Ref	

RRR = Relative risk ratio.

## Data Availability

All original data are available in the Department of Family Medicine, King Abdulaziz University, Jeddah, Saudi Arabia.

## Supplementary Materials

The following are available online at https://www.mdpi.com/article/10.3390/vaccines9070798/s1, Table S1: Reasons for vaccine hesitancy among participants who answered “no” regarding their intentions to get vaccinated (*n* = 619), Table S2: Reasons for vaccine hesitancy among participants who answered “unsure” regarding their intentions to get vaccinated (*n* = 817).
