# Supplementary material for: Attitudes toward the SARS-CoV-2 Vaccine: Results from the Saudi Residents’ Intention to Get Vaccinated against COVID-19 (SRIGVAC) Study
Source: Vaccines (Basel). 2021 Jul 18;9(7):798. doi: 10.3390/vaccines9070798 (PMC8310025; doi:10.3390/vaccines9070798)
Supplement: Supplementary file 1 [file vaccines-09-00798-s001.zip › vaccines-1258604-supplementary.pdf]

Table S1: Reasons for vaccine hesitancy among participants who answered "no" regarding their intentions to get vaccinated (n=619).

	**Strongly Disagree**		**Disagree**		**Neutral**		**Agree**		**Strongly agree**	
	**n**	**%**	**n**	**%**	**n**	**%**	**n**	**%**	**n**	**%**
**Vaccine-related side effects**	9	1.50%	34	5.50%	140	22.60%	205	33.10%	231	37.30%
**Short periods of clinical testing**	9	1.50%	31	5.00%	111	17.90%	151	24.40%	317	51.20%
**Poor efficacy**	18	2.90%	50	8.10%	220	35.50%	135	21.80%	196	31.70%
**Low likelihood of getting the COVID-19 infection**	20	3.20%	83	13.40%	183	29.60%	136	22.00%	197	31.80%
**Lack of trust in health and scientific authorities**	48	7.80%	141	22.80%	198	32.00%	106	17.10%	126	20.40%
**Lack of trust in vaccine outcomes which are produced by pharmaceutical companies**	57	9.20%	118	19.10%	202	32.60%	90	14.50%	152	24.60%
**Pharmaceutical companies promote the vaccine for financial gain**	43	6.90%	118	19.10%	186	30.00%	104	16.80%	168	27.10%
**COVID-19 is harmless**	33	5.30%	133	21.50%	193	31.20%	123	19.90%	137	22.10%
**Preference for acquired immunity via COVID-19 infection**	35	5.70%	63	10.20%	211	34.10%	151	24.40%	159	25.70%
**Vaccines contradict natural remedies**	29	4.70%	99	16.00%	203	32.80%	135	21.80%	153	24.70%
**Moral opposition to get vaccinated**	47	7.60%	122	19.70%	227	36.70%	81	13.10%	142	22.90%

Table S2: Reasons for vaccine hesitancy among participants who answered "unsure" regarding their intentions to get vaccinated (n=817).

	**Strongly Disagree**		**Disagree**		**Neutral**		**Agree**		**Strongly agree**	
	**n**	**%**	**n**	**%**	**n**	**%**	**n**	**%**	**n**	**%**
**Vaccine-related side effects**	27	3.30%	95	11.60%	472	57.80%	169	20.70%	54	6.60%
**Short periods of clinical testing**	13	1.60%	83	10.20%	400	49.00%	223	27.30%	98	12.00%
**Poor efficacy**	45	5.50%	176	21.50%	491	60.10%	76	9.30%	29	3.50%
**Low likelihood of getting the COVID-19 infection**	58	7.10%	193	23.60%	420	51.40%	103	12.60%	43	5.30%
**Lack of trust in health and scientific authorities**	134	16.40%	279	34.10%	339	41.50%	46	5.60%	19	2.30%
**Lack of trust in vaccine outcomes which are produced by pharmaceutical companies**	134	16.40%	230	28.20%	376	46.00%	53	6.50%	24	2.90%
**Pharmaceutical companies promote the vaccine for financial gain**	124	15.20%	218	26.70%	387	47.40%	53	6.50%	35	4.30%
**COVID-19 is harmless**	99	12.10%	263	32.20%	367	44.90%	74	9.10%	14	1.70%
**Preference for acquired immunity via COVID-19 infection**	45	5.50%	135	16.50%	425	52.00%	152	18.60%	60	7.30%
**Vaccines contradict natural remedies**	55	6.70%	200	24.50%	422	51.70%	104	12.70%	36	4.40%
**Moral opposition to get vaccinated**	127	15.50%	252	30.80%	379	46.40%	38	4.70%	21	2.60%
